# TMEM135 is a Novel Regulator of Mitochondrial Dynamics and Physiology with Implications for Human Health Conditions

**DOI:** 10.3390/cells10071750

**Published:** 2021-07-11

**Authors:** Heather K. Beasley, Taylor A. Rodman, Greg V. Collins, Antentor Hinton, Vernat Exil

**Affiliations:** 1 Department of Molecular Physiology and Biophysics, Vanderbilt University, Nashville, TN 37232, USA; heather.k.beasley@vanderbilt.edu (H.K.B.); arabia@usf.edu (T.A.R.); 2 Fraternal Order of Eagles Diabetes Research Center, Iowa City, IA 52242, USA; greg-collins@uiowa.edu; 3 Department of Pediatrics-Cardiology, Carver College of Medicine, University of Iowa, Iowa City, IA 52242, USA

**Keywords:** TMEM135, fission, mitochondrial dynamics, aging

## Abstract

Transmembrane proteins (TMEMs) are integral proteins that span biological membranes. TMEMs function as cellular membrane gates by modifying their conformation to control the influx and efflux of signals and molecules. TMEMs also reside in and interact with the membranes of various intracellular organelles. Despite much knowledge about the biological importance of TMEMs, their role in metabolic regulation is poorly understood. This review highlights the role of a single TMEM, transmembrane protein 135 (TMEM135). TMEM135 is thought to regulate the balance between mitochondrial fusion and fission and plays a role in regulating lipid droplet formation/tethering, fatty acid metabolism, and peroxisomal function. This review highlights our current understanding of the various roles of TMEM135 in cellular processes, organelle function, calcium dynamics, and metabolism.

## 1. The Structure and Function of Transmembrane Proteins

Transmembrane proteins (TMEMs) are essential for cellular structure and function [1,2]. Characterized by their protrusion through a membrane, TMEMs are generally composed of three domains with hydrophilic (extracellular and intracellular domains) and hydrophobic (bilayer domain) properties. The residues of the hydrophobic domain form a coil or helix that spans the lipid bilayer. Although biological membranes are fluid, TMEMs do not change their orientation within the membrane to perform their functions. Acting as membrane linkers, TMEMs instead undergo conformational changes to convey signals to secondary messenger systems. For example, some TMEMs have structures on their extracellular domain that are capable of binding with specific hormones in the extracellular environment [3,4]. Once a hormone molecule is bound, a conformational change at the binding site results in structural changes in the intracellular domain of the TMEM. These changes initiate a cascade of intracellular events that constitute a response to the external environment. 

In addition to allowing cells gather information about the external environment, some TMEMs help control the transfer of solutes across membranes. These transmembrane transporters appear in clusters that create pores or channels within the membrane that can open and close under different conditions or in response to regulatory signals. For example, voltage-gated channels open and close in response to changes in the electrical potential across the membrane, whereas ligand-gated channels open and close in response to binding by specific signaling molecules or substrates [5,6,7,8,9,10]. A number of transmembrane transporters couple the inward movement of one solute to the outward movement of another [11]. Misfolding of transmembrane transporters is associated with a variety of clinical conditions [1,2,12,13]. 

There are several TMEMs in the mitochondria, including two well-studied TMEMs; TMEM70 and TMEM242. TMEM 70 is localized in the inner membrane of the mitochondria and functions as a facilitator of mammalian F1Fo ATP synthase [14,15,16]. Given the known role of TMEM70, mutations in TMEM70 lead to oxidative phosphorylation (OXPHOS) deficiencies linked to many mitochondrial diseases that present as neonatal mitochondrial encephalo-cardiomyopathy in humans [14,15,17,18,19]. Likewise, TMEM242 affects the arrangement of ATP synthase [20], whereas deletion of both TMEM70 and TMEM242 prevents the assembly of ATP synthase, thereby affecting complex I [20].

## 2. The Discovery of Transmembrane Protein 135 (TMEM135)

Very long-chain acyl-CoA dehydrogenase (VLCAD) is an enzyme that catalyzes the first step in the mitochondrial beta-oxidation of certain fatty acids. VLCAD deficiency is a well-documented condition in which pathogenic mutations in the *ACADVL* gene lead to severe physiological consequences, including cardiomyopathy, skeletal myopathy, encephalopathy, and sudden death in children and young adults [21,22,23,24,25,26]. TMEM135 was found to be elevated in the VLCAD-deficient mice. The VLCAD-deficient mouse model recapitulates the clinical phenotypes seen in VLCAD-deficient children. VLCAD-deficient mice display upregulation of critical regulators of mitochondrial biogenesis, such as peroxisome proliferation-activated receptor gamma coactivator-1 alpha (PGC-1α) and acyl-CoA synthase, an enzyme important for fatty acid biosynthesis and sarcolemmal fatty acid uptake [26]; TMEM135 was also elevated in VLCAD-deficient mice. Subsequent studies to further characterize TMEM135 revealed a function in adipogenesis and osteoblastogenesis [27]. The involvement in VLCAD deficiency suggests that TMEM135 may play a role in the regulatory feedback that controls mitochondrial fat metabolism. Further work in VLCAD-deficient mice and *Caenorhabditis elegans (C. elegans)* demonstrated that the metabolic role of TMEM135 in the enhancement of fat storage and mitochondrial function may link TMEM135 to other genetic networks, including insulin signaling [28,29,30,31,32,33,34,35].

## 3. Structural Organization of the TMEM135 Gene and Protein

**Figure 1:**
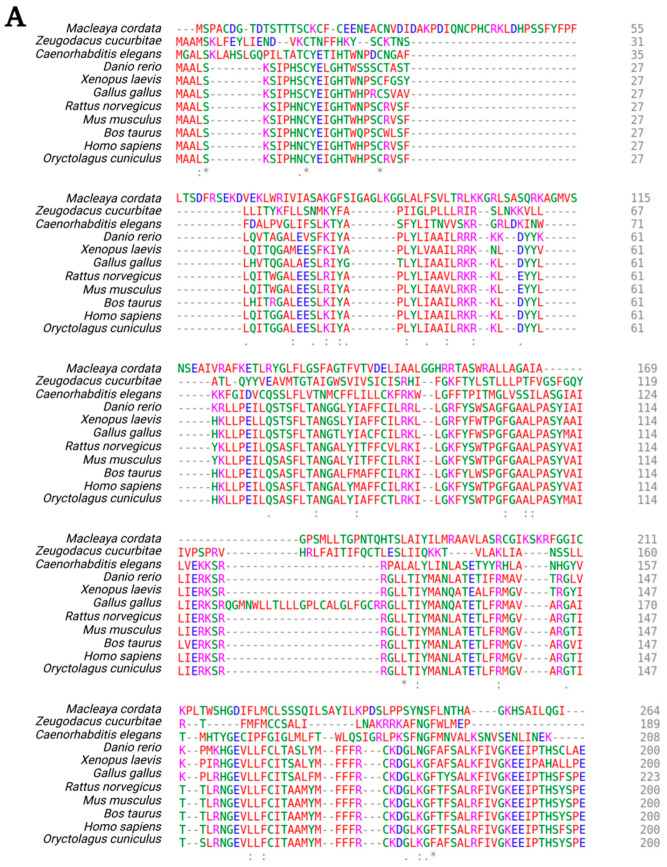

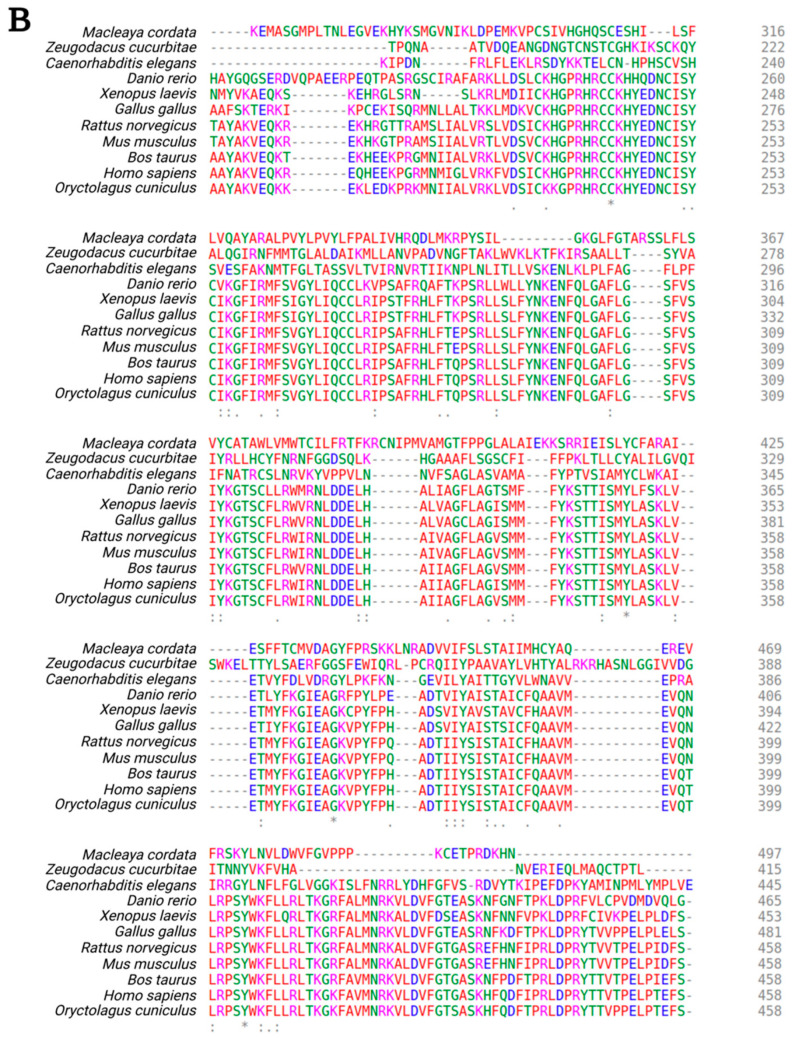
Comparison of the sequence homology of TMEM135 (**A**,**B**) across 11 different species using Clustal Omega Multiple Sequence alignment software (v.1.2.4). Clustal Omega designates the following colors for amino acid groups: AVFPMILW-Red: Small (small + hydrophobic [includes aromatic –Y])DE-Blue: Acidic, RHK-Magenta: Basic -H,STYHCNGQ -Green: Hydroxly + sulfhydryl + amine + G Others-Gray: Unusual amino/imino acids etc. (Accession numbers for TMEM135 species: OUZ99344.1, JAD00441.1, NP_508800.2, NP_001082887.1, NP_001085541.1, XP_040514949.1, NP_001013918.1, NP_082619.3, AAI03394.1, NP_075069.3, and XP_002708692.1 ) [36]. Figure created with BioRender.com (accessed on 15 June 2021).

The gene encoding TMEM135, also named peroxisomal protein 52 (PMP52) (https://www.ncbi.nlm.nih.gov/gene/65084 accessed on 15 June 2021), is located on chromosome 11 in humans (11q14.2) and chromosome 7 in mice. TMEM135 contains six alpha-helical transmembrane domains spanning amino acid positions 67–89, 96–115, 147–169, 300–322, and 332–354, respectively [11,12]. There are two predicted TMEM135 isoform products of alternative splicing. Isoform 1, the canonical sequence, is 458 amino acids in length and has a molecular weight of 52,291 kDa. The shorter isoform 2, which is missing amino acids 133–154 from the canonical sequence, is 436 amino acids in length and has a molecular weight of 49,914 kDa. There are also two additional predicted isoforms containing a total of 330 and 319 amino acids, respectively. The four TMEM135 isoforms of *homo sapiens* are highly conserved across species, with a high degree of homology in *Mus musculus*, *Rattus norvegicus*, *Bos taurus*, *Xenopus laevis*, *Macleaya cordata, Zeugodacus cucurbitae, Danio rerio, Gallus gallus, Oryctolagus cunuculus,* and *C. elegans* (Figure 1).

**Figure 2:**
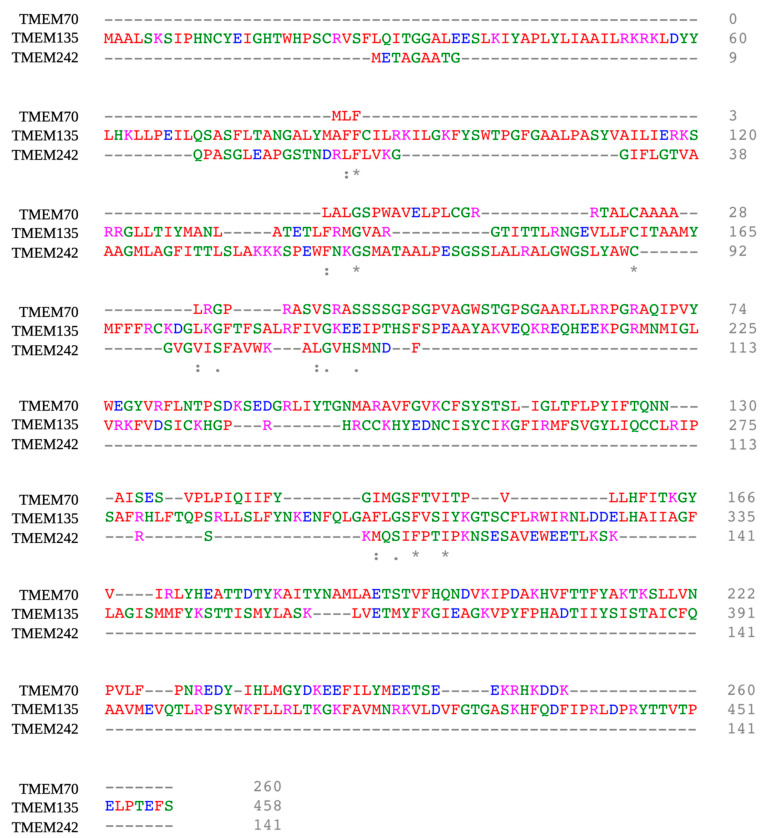
Comparison of the conserved sequence homology of TMEM135 (*homo sapien*) [NP_075069.3] with TMEM70 (*homo sapien*) [AAH02748.2] and TMEM242 (*homo sapien*) [NP_060922.2] using Clustal Omega Multiple Sequence alignment (v.1.2.4.) [36]. Clustal Omega designates the following colors for amino acid groups: AVFPMILW-Red: Small (small + hydrophobic [includes aromatic –Y])DE-Blue: Acidic, RHK-Magenta: Basic -H,STYHCNGQ -Green: Hydroxly + sulfhydryl + amine + G Others-Gray: Unusual amino/imino acids, etc. Figure created with BioRender.com (accessed on 15 June 2021).

Although TMEM135 is a transmembrane protein, it is unclear whether TMEM135 functions as a transmembrane channel. TMEM135 has yet to be crystallized, but the full-length wild-type protein is predicted to have its N-terminus on the outside of the membrane lipid bilayer and its C-terminus on the inside. However, one study in an N-ethyl-N-nitrosourea (ENU)-induced mutant mouse line (FUN025) demonstrated that a point mutation (T > C) in the splice donor site adjacent to exon 12 altered the carboxy terminus, leading to a reverse orientation of the protein across the lipid bilayer [12]. Using the Clustal Omega multiple sequence alignment software (v1.2.4) [36], we aligned the *homo sapien* isoform 1 of the TMEM135 protein sequence with other mitochondrial TMEMs (Figure 2).

## 4. TMEM135 is a Regulator of Mitochondrial Dynamics

The mitochondrion is the only organelle in animals that contains its own self-replicating genome. Mitochondrial DNA encodes 13 essential components of the oxidative phosphorylation (OXPHOS) system, although many mitochondrial proteins are encoded by the nuclear genome. Once thought to be rigid structures, mitochondria migrate through the cell to fuse, divide, and to undergo regulated turnover [37]. Mitochondrial dynamics include the movement of mitochondria along the cytoskeleton and changes in mitochondrial morphology, distribution, and connectivity, which are mediated by tethering, fusion, and fission events [36,37,38,39,40,41,42]. At steady state, fission and fusion events are balanced to maintain mitochondrial morphology and function [39]. When mitochondria undergo fusion, GTPases, mitofusin 1 (MFN-1), and mitofusin 2 (MFN-2) regulate fusion of the outer mitochondria, whereas optic atrophy 1 (OPA-1) regulates fusion of the inner membranes [39,40,41,42,43,44,45,46,47,48]. Mitochondrial fission is mediated by the cytosolic dynamin family member dynamin-related protein 1 (DRP1) [43,44,45], which is recruited from the cytosol to form spirals around mitochondria that constrict and sever the inner and outer membranes.

TMEM135 has an indirect, yet integral role, in mitochondrial metabolism and membrane potential, where it is thought to regulate mitochondrial fission and fusion [6,37,38,45,46,47,48,49,50,51]. Disruption of TMEM135 function in mice can tip the fusion–fission balance towards fusion, leading to an increase in the size and a decrease in the number of mitochondria in cells [12,52]. Lee et al. demonstrated that TMEM135 colocalizes with oligomerized DRP1 and proposed that TMEM135 acts as a regulator of mitochondrial fission by activating DRP1 [12]. In *C. elegans*, overexpression of TMEM135 increases mitochondrial fragmentation and membrane potential, whereas loss of TMEM135 decreases the mitochondrial membrane potential and the rate of oxygen consumption [11]. However, the definitive role of TMEM 135 in oxidative phosphorylation and mitochondrial dynamics continues to be poorly understood.

## 5. TMEM135 and Peroxisomal Transport

Faust et al. first described TMEM135 as a peroxisomal protein in *Drosophila melanogaster* (fly base isoform CG11737) [53]. TMEM135 is a target of the liver X transcription factor in human liver cells and has homology with the Tim17 family of proteins, which mediate protein translocation across mitochondrial membranes [53]. Loss of TMEM135 in hepatocytes reduces concentrations of peroxisomal matrix enzymes that help break down long-chain fatty acids (LCFAs). Despite these findings, little is understood about how TMEM135 is involved in fatty acid beta-oxidation and enzyme transport in peroxisomes. Generally, mitochondria favor the oxidation of short-chain and medium-chain fatty acids (<C12) [54], although palmitate (C16) is the preferred substrate for fatty acid oxidation in the myocardium. Mitochondria and peroxisomes both oxidize LCFAs (C14–C18), whereas only peroxisomes oxidize very-long-chain fatty acids (VLCFAs, >C20) [54]. Renquist et al. used electrophoretic mobility shift assay (EMSA) and chromatic immunoprecipitation (ChIP) analysis to demonstrate that the human TMEM135 promoter contains a liver X receptor (LXR) response element that binds LXRs and mediates LXR-induced transcription [12,55]. This response element was notably not found in murine cells [56]. Furthermore, in human HepG2 cells, decreased expression of TMEM135 caused triglyceride accumulation regardless of diminished lipogenic gene expression, suggesting a potential role for TMEM135 in beta-oxidation. 

To determine its physiological importance, TMEM135 was knocked down via siRNA in the livers of fed and fasted C57BL/6 mice. Consistent with increased fatty acid uptake and beta-oxidation, fasting augmented hepatic fatty acid and NADH concentrations in control mice. Compared with the control mice, fasted TMEM135-knockdown mice displayed a further increase in hepatic fatty acid concentrations and a significant decrease in NADH concentration, suggesting impairment of peroxisomal beta-oxidation [55]. The peroxisomal contribution to overall LCFA beta-oxidation becomes greater during physiological states of increased fatty acid load, such as fasting, which might partly explain why TMEM135 protein levels were increased in heart and skeletal muscle during fasting and cold stress in mice [1]. The observed increases in linoleic acid and total fatty acid levels in fasted TMEM135-knockdown mice are consistent with an impairment of beta-oxidation. Despite some evidence suggesting that TMEM135 localization in the peroxisome may signal peroxisome impairment, there is no known role for TMEM135 in mitochondrial biogenesis, impairment, and/or beta-oxidation [9]. Further analysis of the hepatic NADH and ketone concentrations is needed to confirm peroxisomal beta-oxidation. Renquist et al. demonstrated that TMEM135 mRNA expression is induced by peroxisome proliferation-activated receptor (PPAR) agonists and that the TMEM135 promoter is bound by PPAR [55], indicating that TMEM135 is also a PPAR target gene. These findings suggest that TMEM135 may be a potential therapeutic target in the treatment of age-related diseases associated with peroxisome dysfunction. 

## 6. Potential Physiological Roles of TMEM135

In *C. elegans* and mice, TMEM135 is ubiquitously expressed in a variety of tissues, with the highest expression found under conditions of cold and fasting stress [57,58]. In *C. elegans*, TMEM135 is involved in fat storage and longevity regulation [11]. TMEM135 is expressed in the nucleus, sarcoplasmic reticulum, and plasma membrane, localizing with lipid droplets, peroxisomes, and mitochondria [11,12,32,49,55]. TMEM135 can also be found within mitochondrial endoplasmic reticulum contact sites (MERCs) [11,52,53,55]. MERC sites are specialized contact sites that are thought to be enriched with proteins involved in mitochondrial calcium (Ca^2+^) flux, lipid transfer, and morphology [59,60,61]. Therefore, not only can TMEM135 participate in fission, similar to DRP1 [62], but TMEM135 may also be involved in lipid transport across the mitochondrial membrane within MERC sites [27,63,64]. Any fluctuation in MERC sites that TMEM135 directly or indirectly regulates may serve as a mechanistic link between TMEM135 defects and disruption of cell metabolism. 

**Figure 3:**
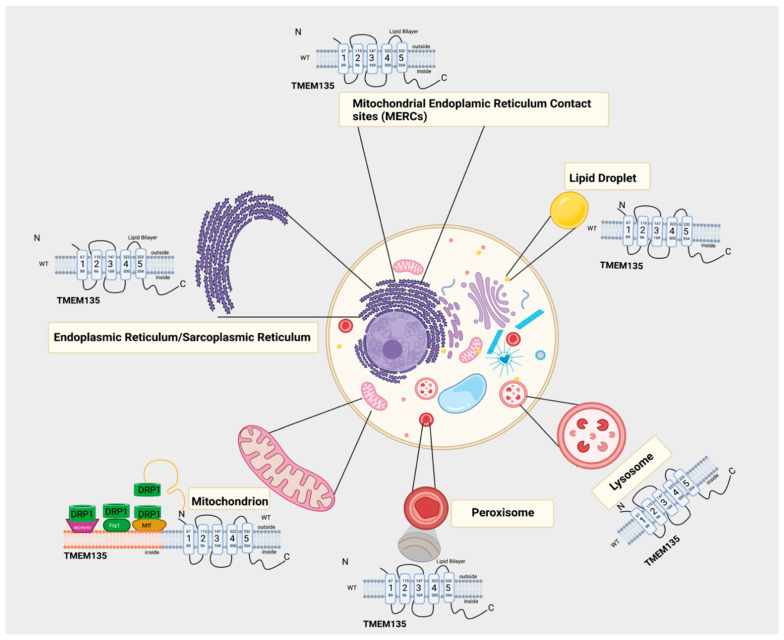
This plausible model of TMEM135 demonstrates the many interactions for TMEM135 in several organelles, including the mitochondria [11] and the mitochondrial endoplasmic reticulum contact sites (MERCs). Additionally, TMEM135 is also a peroxisomal protein [66]. TMEM135 has been shown to co-localize with DRP1 as referenced in Wei Lee et al., 2016 [12]. Here, we show the plausible interaction between TMEM135 and DRP1 in the mitochondria and TMEM135 in the peroxisome [53], lysosome [66], lipid droplets [11], and the endoplasmic reticulum/ sarcoplasmic reticulum (ER/SR) [11]. Figure created with BioRender.com (accessed on 15 June 2021).

**Figure 4:**
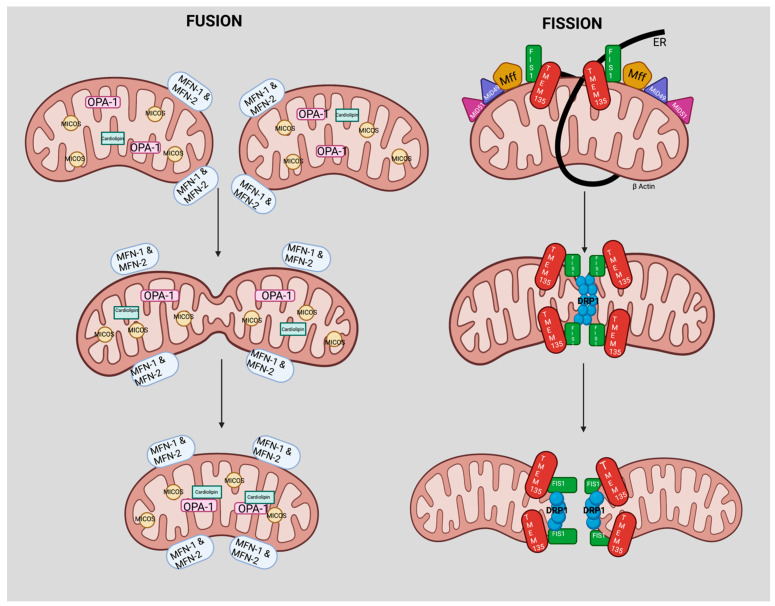
We hypothesize a role of TMEM135 in fission due to Ca^2+^ influx into the mitochondria and stimulation of DRP1-dependent mitochondrial fission. Mitochondrial dynamics are split into two processes: Fusion and Fission. Mitochondrial fusion is coordinated by Mitofusin 1 (MFN-1) and Mitofusin 2 (MFN-2) [light blue color], Optic Atrophy 1 (OPA-1) [light pink], Mitochondrial contact site and cristae organizing system (MICOS) [light yellow], and Cardiolipin [light green]. Mitochondrial fission proteins are coordinated by Mitochondrial Fission 1 protein (FIS1) [green], Mitochondrial dynamic protein of 51 kDa homolog (MiD51) [magenta] and Mitochondrial Fission Factor of 49kDA homology (MiD49) [purple], Mitochondria fission factor (Mff) [dark yellow], Transmembrane Protein 135 (TMEM135) [red] on the outer membrane of the mitochondria; whereas, Dynamin-1-like protein 1 (DRP1) [blue] is located in the inner membrane of the mitochondria. The endoplasmic reticulum (ER) [black] is represented in the fission process. It cannot be ruled out that TMEM135 plays a role in regulating the balance between mitochondrial fusion and fission since it has been proposed that TMEM135 activates DRP1 [12]. Figure created with BioRender.com (accessed on 15 June 2021).

As shown in our schematic (Figure 3), we hypothesize a role of TMEM135 in fission. Breckenridge et al. showed that Ca^2+^ influx into the mitochondria stimulates DRP1-dependent mitochondrial fission and a subsequent release of cytochrome c release [65]. It cannot be ruled out that TMEM135 plays a role in regulating the balance between mitochondrial fusion and fission since it has been proposed that TMEM135 activates DRP1 [12]. Additional investigation is necessary to define the role of TMEM135 in fission (Figure 4). We have summarized the potential physiological roles of TMEM135 in Table 1.

## 7. Potential Role of TMEM135 as a Regulator of Calcium Dynamics

The mitochondria and endoplasmic reticulum (ER) play essential roles in maintaining Ca^2+^ homeostasis and lipids. In addition to storing Ca^2+^, mitochondria can accumulate large amounts of Ca^2+^ to maintain mitochondrial energy metabolism [73,74]; therefore, it is essential to investigate the function of TMEM135 within these homeostatic mechanisms. As mentioned earlier, the mitochondria are linked to the ER by MERCs, which enable mitochondria and ER to exchange Ca^2+^ [74,75]. Any dysregulation or modulation of Ca^2+^ signaling and flux can affect critical cellular networks and structures, including MERC sites [76,77,78]. Notably, Ca^2+^ functions directly and as a second messenger in almost every physiological process—especially in the mitochondria. It should also be noted that Ca^2+^ regulates several cellular processes, including apoptosis, signal transduction, and transcriptional regulation [79,80,81]. Given TMEM135 is involved in mitochondrial dynamics [12,82,83], we speculate that TMEM135 may also regulate Ca^2+^ flux, Ca^2+^ uptake, and Ca^2+^-dependent transcription factors and kinases. TMEM135 may regulate several transcription factors that have integral roles in maintaining Ca^2+^ dynamics in the cell.

*C. elegans* studies revealed that TMEM135 could also regulate Forkhead box O (FOXO), FoxO, expression [11,84]. Elevated nuclear expression of FoxO and its target genes can contribute to muscle wasting and cell death [84]. FoxO transcription factors can also contribute to cardiac growth, cardiac remodeling, and cardiac phenotypes in laminopathies, diabetic cardiomyopathy, and ischemia-reperfusion injury [16,17,18]. Several cellular responses related to stress and aging are downstream of FoxO [85,86]. The expression of TMEM135 is connected to several stress-induced signaling pathways, including the p38 pathway [87]. Upstream of FoxO, the p38 signal transduction pathway mediates FoxO translocation to the nucleus [87]. The c-Jun N-terminal kinase is also a positive regulator of FoxO that mediates FoxO translocation to the nucleus [85,86]. Interestingly, c-Jun is a Ca^2+^-dependent kinase along with ATF4, an isoform of CREB. Fusakio et al. showed that ATF4 enhances the transcription of genes involved in oxidative stress, ER stress, and mitochondrial stress [88]. Because TMEM135 appears critical for FoxO regulation [11], it might have clinical relevance for aging and heart-failure biology, beyond fatty-acid beta-oxidation defects. 

Despite the lack of a current structure for TMEM135, several proteins have experimental and predicted interactions with TMEM135 that have roles in calcium signaling, including Sphingomyelin phosphodiesterase (SMPD3) [89,90]. Detected experimentally by affinity chromatography assay [89,90], TMEM135 and SMPD3 share an association (https://string-db.org/network/9606.ENSP00000306344, Saccessed 1 July 2021). It is well understood that sphingolipids are integral parts of lipid membranes [91]. More importantly, sphingolipids can activate or inhibit channels and modulate calcium signaling [92]. Interestingly, the function of SMPD3 is to hydrolyze sphingomyelin to form ceramide and phosphocholine [93,94,95]. SMPD3 has a crystallized structure (5UVG) that has two calcium ion ligands [96,97]. Given the localization TMEM135 has with lipid droplets and the experimental evidence of interaction and shared homology with SMPD3, we postulate that TMEM135 has a role in altering Ca^2+^ dynamics. 

## 8. General Characteristics and Profiling of TMEM135 in Human Diseases

Many TMEM proteins contribute to oncogenesis, including TMEM135 [70,97]. In humans, TMEM135 was identified as an apoptosis-regulating protein in BRCA1-mutant estrogen receptor-positive breast cancer [27,55,62,70]. Natrajan et al. performed a sequencing analysis of independent hereditary *BRCA1* and non-*BRCA1* breast cancers cases and identified *TMEM135* as a potential driver of breast cancer [98]. TMEM135 mutations were also identified in melanoma patients and in recurrent gene fusions associated with several other cancers [99,100].

TMEM135 is highly expressed in brain tissue. A study by Franic et al. suggested an association between TMEM135, learning, and intelligence [101]. In mice, the pathogenic FUN25 mutation in TMEM135 was associated with age-dependent pathologies, including accelerated retinal aging reminiscent of human macular degeneration; however, mutations in human TMEM135 have not been reported in macular degeneration patients [12].

Human interactome studies looking at networks of protein–protein interactions suggest that TMEM135 interacts with proteins involved in lipid synthesis, cholesterol-binding, cholesterol transport, membrane rafting, and Ca^2+^ modulation, including sphingomyelin phosphodiesterase 3 (SMPD3), which has a critical role in ceramide synthesis [102,103,104]. TMEM135 was also found to be differentially expressed (with a high degree of ethnic difference) in subcutaneous adipose tissue between insulin-resistant and insulin-sensitive individuals when matched for body mass index [57].

Genome-wide association studies revealed that TMEM135 is involved in bone density maintenance and osteoporosis [64]. Furthermore, large and rare copy-number variations in TMEM135 were associated with moderate to extreme obesity [58]. TMEM135 is also differentially expressed in peripheral blood mononuclear cells of patients after treatment with a high-dose statin, a cholesterol-lowering medication, in the YELLOW II Study, indicating a potential role in inflammation and/or cholesterol flux capacity [37]. Chu et al., demonstrated that when TMEM135 was knocked down, the plasma membrane cholesterol levels were significantly reduced, suggesting that TMEM135 has a unique and integral role in lysosome-peroxisome membrane contacts [66]. Further studies reported that perturbed intracellular cholesterol distribution imposed by lysosomal cholesterol accumulation during TMEM135 depletion, is closely associated with impaired ciliogenesis [69]. TMEM135 depletion prevents ciliary vesicle elongation, a characteristic of impaired Rab8 function [69]. In addition, TMEM is also involved in chemically induced hepatic steatosis (non-alcoholic fatty liver disease) [105] and hypertrophic cardiomyopathy [106]. Forced overexpression TMEM135 in mouse hearts led to a form of cardiomyopathy characterized by hypertrophy, increased collagen deposits, and premature cardiac aging [20].

## 9. Perspective

**Figure 5:**
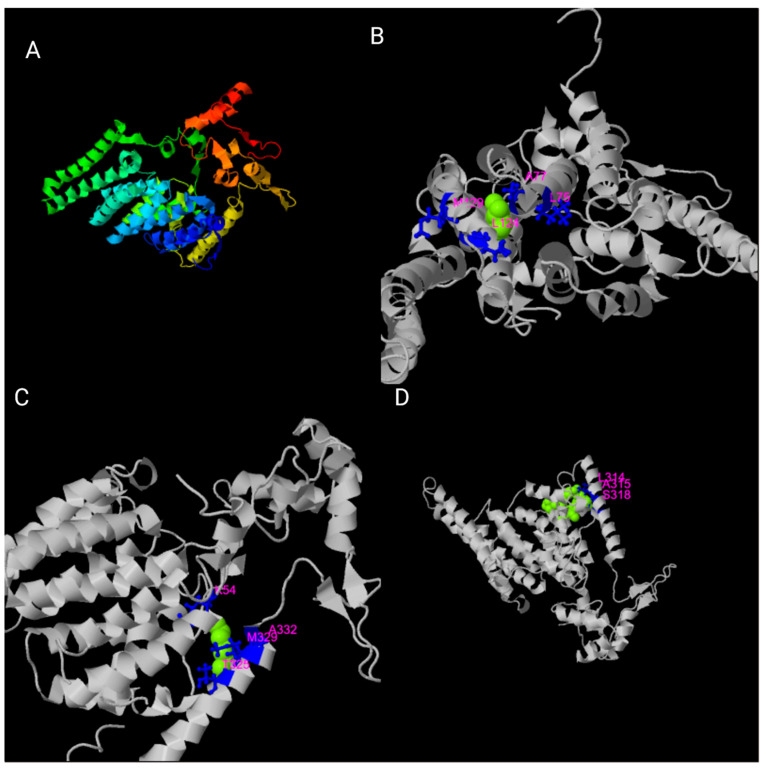
Predicted ligands for TMEM135 using I-TASSER software [71,72,107]. (**A**) The different colors represent the different alpha helixes. (**B**–**D**) I-TASSER (version 5.1) software provides biological annotations of the target ligand by COACH based on the I-TASSER structure prediction, represented by the magenta-colored amino acid residues. Figure created with BioRender.com (accessed on 15 June 2021).

The role of TMEM135 in mitochondrial dynamics has implications for adipogenesis, mitochondrial function, and fat storage. Previous reports suggest a critical link between TMEM135 and aging; however, there is still no definitive insight into the role of TMEM135 in aging outside of phenotypic observations linked to polymorphisms in the *TMEM135* gene [12]. A TMEM135 crystal structure could delineate the exact position of TMEM135 in mitochondrial metabolism and dynamics, as well as the cellular physiology and biophysical characteristics of the protein. Using I-TASSER (Iterative Threading ASSEmbly Refinement) software (version 5.1) [71,72,107], we have determined potential ligands, including oleic acid, chlorophyll, and derivatives of glucose, to interact with TMEM135 (Figure 5). 

Due to the lack of characterization of the electrical and biophysical properties of TMEM135, it will be helpful to use biophysical techniques such as patch clamping to determine which agonist(s) activates TMEM135. Additionally, a better understanding of the activation of TMEM135, e.g., activation by mechanical force, would prove helpful. Interestingly, it has been well established that ion channels can change the cell membrane potential [5,108,109]. Therefore, further investigation is needed to understand how TMEM135 can change the membrane potential. One way to experimentally test this is by overexpressing TMEM135 in a well-characterized cell line such as HEK293 or CHO cells and assessing biophysical changes. 

Further research into the precise subcellular localization of TMEM135 would be beneficial for understanding the functions of TMEM135. Additionally, the activity-regulating ligands and kinases of TMEM135 have yet to be identified. TMEM135 may serve as a target for future anti-aging therapeutics, but further study is required. Creating a conditional loss-of-function mouse system would be beneficial to understanding the effects of TMEM135 ablation and overexpression in the tissue of interest. Overall, TMEM135 has been implicated to be a novel regulator of mitochondrial dynamics and cell physiology [6,37,38,45,46,47,48,49,50]; thereby, making TMEM135 a critical piece in understanding health and disease.

## Figures and Tables

**Table 1:** TMEM135 has many implications in disease and many potential physiological roles. Using The Human Protein Atlas Databank [67] and literature, we show the predicted locations of TMEM135, expression in human tissue, known interactions with organelles and vesicles, the physiological role of TMEM135 [68], and the implication of TMEM135 in human disease.

Predicted Location	Major Expression in Tsue	Known Interactions with Organelles/Vesicles	Physiological Role	Implication in Human Disease
Vesicles [66] Membrane [66]	Medium expression in: Cerebral cortex, Cerebellum Hippocampus, Caudate Nasopharynx, Bronchus, Lung, Duodenum, Small intestine, Cervix, uterine, and Adipose tissue [66] Low expression in: Thyroid gland, Parathyroid gland, Adrenal gland, Oral mucosa, Salivary gland, Stomach, Colon, Rectum, Liver, Gallbladder, Kidney, Urinary bladder, Testis, Epididymis, Seminal vesicle, Vagina, Ovary, Fallopian Tube, Endometrium, Placenta, Breast, Smooth muscle, Skin, Appendix, and Soft tissue [66]	Lipid Droplets [11] Mitochondria [11] SR/ER [11] Peroxisome [52] Lysosome [65] Proposed that TMEM135 may interact with MERCs	Cholesterol transport; [67,69] Intracellular cholesterol distribution; [67,69] Fat storage and longevity regulation [67,69] Regulation of ciliogenesis (cholesterol dependent) [67,69]	Profiling in Breast cancer prognosis [27,55,62,70] Profiling of TMEM135 in patients to assess inflammation and/or cholesterol flux capacity [37] Profiling of patients with insulin-resistant and insulin-sensitivities when matched for body mass index [56] Profiling of TMEM135 in patients to assess associations with moderate to extreme obesity [57] Profiling of TMEM135 in bone density maintenance and osteoporosis [63] Profiling of TMEM135 in patients to assess non-alcoholic fatty liver disease) [71], hypertrophic cardiomyopathy [72], and premature cardiac aging [20].
